# Quantum transduction from electron spin state to a signaling state in a wild-type LOV photoreceptor

**DOI:** 10.64898/2026.06.30.735402

**Published:** 2026-07-03

**Authors:** William Salvia, Joshua Straub, Shiny Maity, Alexey Bogdanov, Subhajyoti Chaudhuri, Eric Han, Songi Han

**Affiliations:** 1 Department of Chemistry, Northwestern University, Evanston, IL 60208, USA; 2 Department of Chemical and Biological Physics, Weizmann Institute of Science, Rehovot 7610001, Israel

## Abstract

Whether coherent spin dynamics can control biological regulation remains an open question. Here we show that the electron spin state in the wild-type *Avena sativa* phototropin 1 Light-Oxygen-Voltage domain 2 (AsLOV2) modulates FMN–cysteine photoadduct formation, the covalent bond that defines its signaling state. Although AsLOV2 was thought magnetically inactive, we observe a magnetic field effect (MFE) on its fluorescence, emerging above ~100 mT and strengthening to 1300 mT. The MFE establishes a spin-correlated radical pair on the photoadduct pathway, one rendered inaccessible to direct spectroscopy by the same strong coupling that sets the high-field onset. It arises from *g*-factor asymmetry within the closely spaced FMN–cysteine radical pair overcoming its dipolar coupling to drive coherent singlet–triplet interconversion. We furthermore observe an MFE in UV–Vis absorption that, unlike fluorescence, shows the field modulates productive bond formation itself. LOV domains are ubiquitous biological regulators, suggesting that quantum transduction relying on radical pairs as molecular qubits may be a general property among natural and engineered photoreceptors.

## 2 Introduction

A growing body of evidence shows that quantum properties can affect biological function, yet the findings are contested, the mechanisms are disputed, and conclusive cases are few [1, 2, 3]. Open questions include whether such effects are rare or common, and whether quantum coherence can directly modulate protein signaling that underlies important biological processes. The latter has never been demonstrated.

A central challenge for non-trivial quantum effects in biology, those requiring sustained coherence rather than only quantized states, is the fast decoherence of quantum states in warm and crowded solution[1, 2]. Electron spin states are an exception: their coherence can persist on the timescales relevant to biochemical processes, often as spin-correlated radical pairs (SCRPs). An SCRP forms through photoexcitation followed by electron transfer, generating spatially separated radicals with a well-defined initial spin state, triplet or singlet, and thus spin correlation. SCRPs underlie proposed mechanisms in avian magnetoreception and photosynthetic electron transfer[4, 5, 6, 7, 8, 9].

In bird cryptochromes, flavin adenine dinucleotide (FAD) is photoreduced by tryptophan to form an SCRP across >20 Å[5, 10]. Its reactivity depends on singlet vs. triplet spin states described by the radical pair mechanism (RPM): back electron transfer to the FAD ground state proceeds only from the singlet state, so if singlet-triplet interconversion occurs within the coherence time, flavin fluorescence becomes field-dependent. Such magnetic field effects (MFEs) have been observed in cryptochromes [5, 11], photolyases [12, 5, 13], and free flavin solutions with reductants[14]. Whether cryptochrome’s biological signaling derives from this RPM remains unsettled [11].

Light-Oxygen-Voltage (LOV) domains are flavoproteins and ubiquitous in plants, fungi, bacteria, and the human gut microbiome[15, 16, 17]. The LOV2 domain of *Avena sativa* phototropin 1 (AsLOV2) is a blue-light photoreceptor widely used in optogenetics[18, 19, 20]. In AsLOV2, blue light drives covalent bond formation between FMN C4a and the sulfur of cysteine 450 (C450), and this photoadduct has been proposed to form through an RPM[21]. Direct observation of the radical pair has been elusive[21, 22], and wild-type (wt) AsLOV2 shows no MFE at fields up to 25 mT [23], leading to its classification as magnetically inactive. We show this classification to be an artifact of the field range surveyed: the strong coupling and Δg-driven interconversion push the MFE onset above 100 mT, where it had never been sought. The magnetically active flavoproteins studied to date do not have the only mechanism of flavoprotein magnetosensitivity; the generic LOV domain is magnetically active too.

Every flavoprotein in which an MFE has been demonstrated, including avian cryptochrome, engineered AsLOV2 mutant MagLOV, photolyase, and free flavin with added reductant, lack a reactive cysteine and have radical partners far apart (> 15 Å) and weakly coupled. For LOV domains, this is the exception, not the rule. The FMN–cysteine photoadduct motif, in which the radicals sit ~4.4 Å apart and are strongly coupled, is the conserved, canonical arrangement across the LOV family [24].

An exception to the trend among LOV domains is MagLOV, which replaces its C450 with a proline and uses a distant (~15 Å) tryptophan [25] as the electron donor, producing a cryptochrome-like tryptophan-flavin radical pair driven by hyperfine asymmetry[23, 26]. MagLOV displays a large fluorescent MFE, up to 75%[23], and has been demonstrated as an in-cell reporter[23, 26, 27]. MagLOV does not form the covalent FMN-cysteine photoadduct, so its strong MFE is not directly connected to a downstream conformational signaling event (see Results and S.7 for more discussion). Wt AsLOV2 is the opposite case: its FMN-cysteine photoadduct formation and downstream signal transduction are well established[28, 29, 30, 21, 31, 22], yet no MFE has been reported. The link between coherent radical pair spin dynamics and biological signal transduction is therefore missing in both.

In this work we challenge the assumption that AsLOV2 and other LOV domains are magnetically inactive. The FMN and cysteine radicals sit far closer (~4.4 Å) than the FAD-tryptophan pair in cryptochromes [5] and MagLOV (≥15 Å) (M.3)[25], giving an electron dipolar coupling of hundreds of MHz that blocks hyperfine-driven interconversion at low field. But the flavin and cysteinyl radicals have unusually distinct *g*-factors, so at high field the Larmor frequency difference exceeds the electron-electron coupling and drives $T_{0}-S_{0}$ interconversion within the SCRP coherence time. This Δ*g*-driven mechanism resembles those in photosynthetic reaction centers[32, 33] and donor-acceptor bridges[34, 35], but here the radicals are strongly coupled and the pair is directly reactive, forming the FMN-cysteine bond that defines the protein’s signaling state[36, 21, 28, 29].

Because AsLOV2’s photocycle is well-understood, an MFE can be tied directly to downstream biochemical events: photoadduct formation drives the J*α* helix extension[36, 21, 28, 29] that activates engineered effector domains [37] or a drug-binding nanobody[18], so an MFE offers a magnetic handle on the function.

We report an MFE on wild-type AsLOV2 that emerges only at high field, acting directly on chemical bond formation and, by implication, on the subsequent conformational change and signaling state that follow[29]. Fluorescence experiments show a strong field dependence for AsLOV2 and a weak one for MagLOV; UV–Vis experiments with and without a magnetic field reveal an MFE on FMN-cysteine bond formation. We model the field dependence to show how strong electron coupling and *g*-factor asymmetry together produce the observed MFE. These results establish a quantum signal transduction mechanism in a wild-type protein, with implications for quantum effects in biology and for engineering magnetosensitive proteins. Given the ubiquity of LOV domains, FMN-cysteine magnetoreceptors operating at high field may be widespread.

## 3 Results

### 3.1 High-field emergence of AsLOV2 MFE

**Figure 1:**
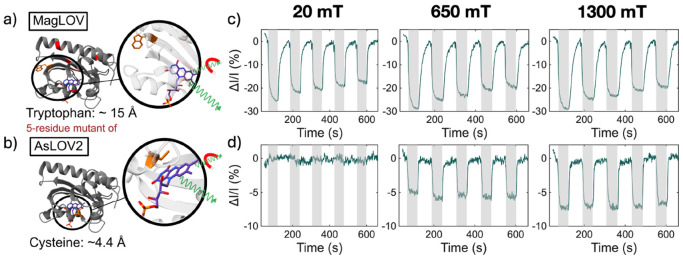
**(a)** A protein structure of MagLOV (PDB 2v1a) with the 5 residues mutated from wild-type AsLOV2 highlighted in red. The tryptophan key for the electron transfer step and the flavin are highlighted in a blow-up subsection. **(b)** A protein structure of AsLOV2 (PDB 2v1a) with the covalently binding cysteine and flavin highlighted in a blow-up subsection. **(c)** MagLOV exhibits a field-independent MFE on fluorescence. In the regions shaded in grey, a magnetic field was turned on. In response to this field, a change in normalized fluorescence is observed. MagLOV shows relatively constant MFEs across fields 20 mT (maximum of 25%), 650 mT (maximum of 30%), and 1300 mT (maximum of 30%). Decreases in the MFE size over an experiment has been observed previously [26] and is due to damage of the MagLOV sample by the laser. **(d)** In contrast, AsLOV2’s MFE uniformly strengthens with increasing field, 20 mT (0%), 650 mT (5%), and 1300 mT (7%). For a full descrption of AsLOV2’s MFE field-dependence see Fig. 3.

We began by measuring the magnetic field dependence of fluorescence under continuous blue-light excitation in both MagLOV and AsLOV2 at fields ranging from 20 to 1300 mT using an electromagnet for field control (setup shown in Fig. S2, processing details in S.1 and Fig. S1). Previous studies had shown a strong fluorescent MFE in MagLOV of up to 75% at fields of 20 mT, with limited field dependence in the range of 10 – 50 mT (descendant MagLOV2 is similar)[38] but no response for AsLOV2 at these fields [23, 26]. This was attributed to the fact that in MagLOV, the nearby cysteine residue is mutated into a proline, so the reducing agent is a distant (~1.5 nm) tryptophan [25] rather than the nearby cysteine as is the case for AsLOV2 (Fig. 1). Our results at 20 mT agreed with these studies, with MagLOV showing a ~25% MFE and AsLOV2 showing no measurable MFE. Looking at fields of 350 and 1300 mT, we found that MagLOV has a relatively field-independent MFE in this range, with a change in fluorescent MFE of only 5% between 20 and 1300 mT (Fig. 1(c). This near-independence is expected for hyperfine-driven systems: once the Zeeman splitting (560 MHz at 20 mT) exceeds the hyperfine difference, $S_{0}-T\pm$ interconversion is suppressed and higher fields have little further effect.

For wild-type AsLOV2, an MFE emerged at $\tilde{1}00$ mT and increased with magnetic field strength: 1% at 150 mT (Fig. 3(d)), 5% at 650 mT, and 7% at 1300 mT (Fig. 1(d); full field dependence in Fig. 3(d)). AsLOV2 therefore exhibits a fluorescent MFE, but only at field strengths well above those previously surveyed for flavoprotein magnetosensitivity. This effect could be readily recapitulated on the benchtop using inexpensive N52-grade neodymium permanent Halbach type magnets that reach above 300 mT (See S.4 and Fig. S4), making the measurements broadly tractable. This strong and high-onset field dependence distinguishes AsLOV2 from other magnetosensitive flavoproteins, including cryptochromes, MagLOV, and photolyases [23, 5, 26, 14, 12, 11].

This high-onset dependence originates in the short FMN–cysteinyl radical separation (4.4 Å), where dipolar and exchange couplings far exceed those of the FMN/FAD–tryptophan radical pairs typical of known magnetosensitive flavoproteins. The same proximity that sets the field threshold also lets the radical pair react directly, forming the FMN-cysteine bond, which is a chemical reactivity absent from previously studied flavoproteins showing magnetosensitivity (mechanism in Sec. 3.3).

### 3.2 Magnetic field modulates bond formation

**Figure 2:**
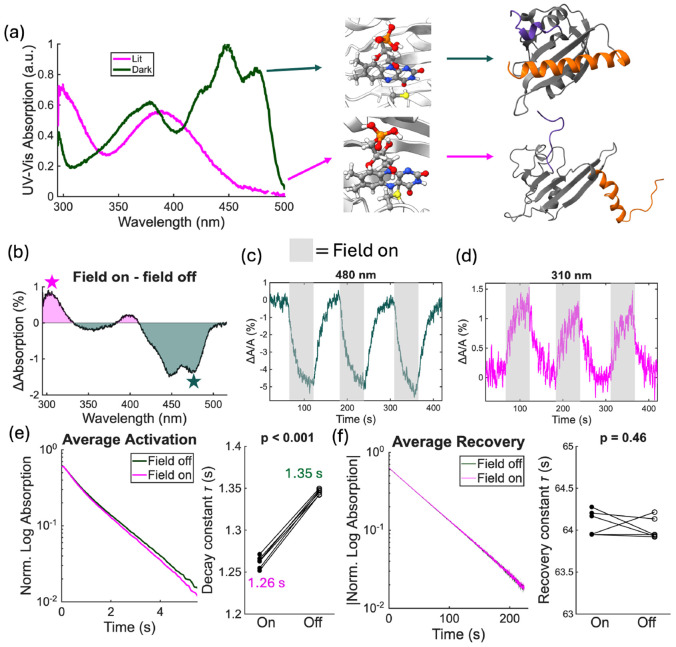
Magnetic field effect on AsLOV2 bond formation. **(a)** UV–Vis absorption spectra for dark and lit AsLOV2 states, linked to cysteine bond formation and conformational response, as shown by AsLOV2 photoactivation. The cysteine dark and lit state structures were generated using quantum chemistry calculations (SI S.10). The cysteine-bound AsLOV2 structure is from pressure-activated simulations published in Maity et al. [29] **(b)** A 650 mT magnetic perturbation of an equilibrium between dark and lit states of AsLOV2, established by incompletely photoexciting an AsLOV2 sample. **(c)** Time-dependent magnetic field effects on the equilibrium absorption depicted in (b) at 480 nm, where the dark state has a higher intensity than the lit state. This represents the consumption of the dark state when the magnetic field is turned on. **(d)** Magnetic field effects on the equilibrium absorption at 310 nm, which represents the lit state population, showing that it increases when the magnetic field is applied. **(e)** Exponential activation from the dark to lit state is sensitive to whether the field is applied, with a significantly different decay constant (p < 0.001). **(f)** The curve showing the recovery of the AsLOV2 sample back to the dark state is not sensitive to whether a magnetic field is applied (p = 0.46).

The direct reactivity in the AsLOV2 radical pair is well-characterized. Previous work identified the cysteine-FMN radical pair as an intermediate in the formation of the covalent C-S bond that produces AsLOV2 signaling state[21, 31]. If the fluorescence MFE we observed arises from this radical pair, then the chemical bond formation step itself should be magnetic field dependent. A fluorescence MFE, while diagnostic of a radical pair, reports only on excited-state branching, that is, on how spin state partitions the radical pair between decay channels; it does not establish that the field changes the amount of covalent product formed. We tested this prediction with UV-visible absorption (UV–Vis) above the field threshold established by the fluorescence results. The dark and lit states of AsLOV2 have well-resolved UV–Vis signatures (Fig. 2(a)) [29]: the dark state shows peaks at 447 and 473 nm and the bound state at 380 nm and < 300 nm, giving a real-time readout of the signaling-state population. We corroborate these UV–Vis signatures using quantum chemical TDDFT modeling (Fig. S11). Using an absorption setup inside the electromagnet pole gap (Fig. S5; Methods), we cycled the field off/650 mT, mirroring the fluorescence protocol.

Application of the 650 mT field produced a UV–Vis lineshape change consistent with increased lit-state population (Fig. 2(b)). To resolve this population shift quantitatively, we monitored two wavelengths with maximum contrast between the dark and lit states and minimized contribution from the other spectral component – 480 nm (dark, unbound state) and 310 nm (lit, bound state) – during repeated cycling on and off of the magnetic field. The 480 nm absorbance decreased under the applied field, indicating a decrease in the population of the dark, unbound state (Fig. 2(c)). Absorption at 310 nm correspondingly increased (Fig. 2(d)). The 650 mT magnetic field, therefore, shifts the equilibrium between the bound and unbound flavin states. By tracking the photoadduct UV–Vis signature, we measure the field effect on product formation itself: the population of the covalent FMN–cysteine bond that defines the signaling state, as opposed to the field effect on fluorescence that reports on the population of fluorescent FMN states. To our knowledge, this is the first demonstration that a magnetic field modulates productive covalent bond formation between radicals in a protein spin-correlated radical pair.

Magnetic field effects on FMN-cysteine bond formation also appears in the activation kinetics. We monitored the absorbance at 480 nm following the onset of blue-light excitation with the field off and with the field on at 650 mT, and fit the data to an exponential decay to extract the activation rate, $k_{cat}$ (Methods M.8). Activation in the 650 mT field was faster than without the external field (i.e. at Earth’s magnetic field) showing $k_{act,on}^{-1}=1.26\pm 0.01\;s$ and $k_{act,off}^{-1}=1.35\pm 0.01\;s$ with a significant *p*-value of *p* < 0.001 across 6 different trials (Fig. 2(e)), consistent with the magnetic field increasing the rate of bond formation by rendering the radical pair spin dynamics more favorable towards bond formation. This rate enhancement corresponds to a 6.7 ± 0.6% kinetic MFE (see Methods M.9), in agreement with the fluorescence MFE observed at the same field (Fig. 1). As a control against systematic artifacts, we measured the recovery kinetics (FMN-cysteine thermal bond cleavage following light shutoff), which does not proceed through a spin-correlated radical pair mechanism. The recovery time constant ($k_{rec}^{-1}$), extracted by exponential fit, showed no significant difference between field-on and off conditions (*p* = 0.46; Fig. 2(f)). At 350 mT, both equilibrium population shifts and activation showed the same field response with a smaller effect, as expected at lower field (Fig. S7). The magnetic field therefore acts specifically on the bond formation pathway through the spin-correlated radical pair mechanism.

### 3.3 g-factor asymmetry (Δg) overcomes strong dipolar coupling at high field

Singlet-triplet interconversion in an SCRP is driven by asymmetry between the two electron radical environments; in the limit of identical environments, only relaxation (not coherent interconversion) occurs. For MagLOV and the avian cryptochrome, this asymmetry arises from differential hyperfine interactions, and the ~1.5 – 2 nm inter-radical distance makes the dipolar coupling weak (~few MHz) and negligible to first order [25, 26]. The low-field hyperfine difference efficiently mixes $S_{0}$ with all three triplet states, and as the field is increased, the Zeeman energy separates the $T_{\pm }$ states from $S_{0}$ and suppresses the hyperfine-driven mixing, producing the conventional low-field MFE.

**Figure 3:**
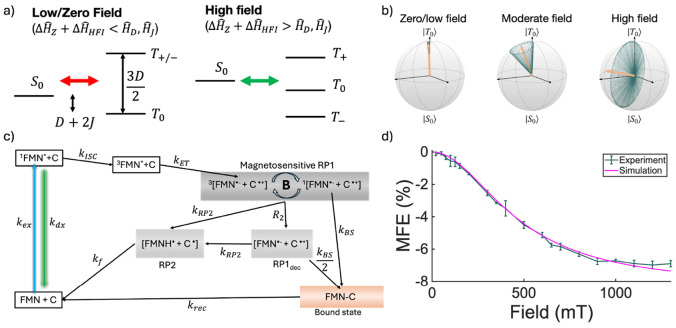
Mechanism behind AsLOV2 magnetic field effects. **(a)** Low and high field regimes (dipolar basis vs Zeeman basis) allowing interconversion only at fields where the asymmetry in the Zeeman interaction exceeds the dipolar interaction. **(b)** Interconversion of $T_{0}$ and $S_{0}$ visualized on a Bloch sphere spanned by $T_{0}$ (up) and $S_{0}$ (down) at three field regimes: low field ($\Delta \omega_{e}<D∕2+J$), moderate field ($\Delta \omega_{e}∼D∕2+J$), and high field ($\Delta \omega_{e}>D∕2+J$). The effective Hamiltonian axis (orange) tilts from the *z*-axis toward the *x*-axis as the field increases, driving precession (green) into states with increasing singlet character. **(c)** AsLOV2 photocycle kinetics to model the AsLOV2 fluorescence MFE. The state $RP1_{dec}$ captures loss of spin correlation through $T_{2}$ relaxation. **(d)** Simulation of fluorescence MFE field dependence using model in (c). Rates and full photocycle description in SI S.8 and rate values in Table 1.

For AsLOV2, the picture is qualitatively different. The inter-radical distance is ~4.4 Å (measured from C450’s sulfur to FMN’s C4a carbon in PDB 2v1a)[30], so that the dipolar coupling constant $d=\frac{-\mu_{0}}{4\pi }\frac{\hbar \gamma_{e}1\gamma_{e}2}{r^{3}}$ of d∼610MHz. This dipolar coupling is significantly larger than the radicals’ hyperfine couplings [39, 40]. This strong dipolar coupling suppresses triplet-singlet interconversion in the low-field limit (Fig. 3(a,b)) , since the low-field eigenstates of the dipolar Hamiltonian are $S_{0}$, $T_{0}$, $T_{+}$, $T_{-}$ (or $T_{x}$ and $T_{y}$, depending on the symmetry of the system). The FMN-cysteinyl radical pair, however, has an unusually large Δg for organic radicals: $g_{iso,FMN}=2.0034$ and $g_{iso,cysteine}=2.035$ [41]. As the magnetic field is increased, the electron Larmor frequency asymmetry $\Delta \omega_{e}=\omega_{e1}-\omega_{e2}$ grows, enabling mixing between $S_{0}$ and $T_{0}$. In AsLOV2 the $T_{\pm }$ states remain significantly separated in energy at both low field (by dipolar coupling) and high field (by Zeeman energy), so their coupling to $S_{0}$ is inefficient regardless of initialization and occupancy. This mechanism resembles the Δg-effect described for radical pairs in donor-acceptor bridges and photosynthetic reaction centers [32, 33, 34, 35, 42, 43, 44], but differs in the strong interradical spin coupling present in AsLOV2. MFEs resulting from the Δg-effect are rarely observed below 1 T, and previously only in radicals associated with a metal ion [43, 42].

We restrict our analysis to the 2D basis spanned by the $S_{0}-T_{0}$ manifold. Within this manifold, the spin Hamiltonian consists of the Zeeman Hamiltonian, ${\hat{H}}_{Z}=\omega_{e1}{\hat{S}}_{1z}+\omega_{e2}{\hat{S}}_{2z}$, the secular dipolar Hamiltonian, ${\hat{H}}_{D}=D(3{\hat{S}}_{1z}{\hat{S}}_{2z}-{\hat{S}}_{1}\cdot {\hat{S}}_{2})$ with $D=d\frac{1-3cos^{2}(\theta )}{2}$, and the exchange Hamiltonian ${\hat{H}}_{J}=-2J\hat{S}\cdot {\hat{S}}_{2}$, yielding

(1) $${\hat{H}}_{S_{0}-T_{0}}=\left(\begin{array}{cc} D∕2+J & \Delta \omega_{e}\\ \Delta \omega_{e} & -(D∕2+J) \end{array}\right)$$

where $\Delta \omega_{e}$ scales linearly with field strength. This form is equivalent to off-resonance Rabi oscillations: the off-diagonal $\Delta \omega_{e}$ mixes $S_{0}$ and $T_{0}$, with the degree of mixing set by the ratio of the off-diagonal elements $\Delta \omega_{e}$ to the diagonal elements D∕2+J. For a $T_{0}$-initialized state, the singlet probability evolves with time t as

(2) $$P_{S}(t)=\frac{\Delta \omega_{e}^{2}}{\Delta \omega_{e}^{2}+{\left(D∕2+J\right)}^{2}}\;\sin^{2}\;\sqrt{\Delta \omega_{e}^{2}+{\left(D∕2+J\right)}^{2}t}$$

with maximum singlet probability ${\Delta \omega }_{e}^{2}∕({\Delta \omega }_{e}^{2}+{\left(D∕2+J\right)}^{2})$ reached when $t=\left(\frac{\pi }{2}+n\pi \right)∕\sqrt{{\Delta \omega }_{e}^{2}∕({\Delta \omega }_{e}^{2}+{\left(D∕2+J\right)}^{2})}$ for integers n. This evolution is shown on the $T_{0}-S_{0}$ Bloch sphere across three field regimes in Fig. 3(b): at high field, the effective Hamiltonian axis tilts toward the $S_{0}-T_{0}$ mixing direction, driving the state into substantial singlet character.

Although AsLOV2 has suppressed singlet-triplet interconversion at low fields and increased interconversion at high fields (the reverse of MagLOV), both proteins show the same negative MFE. This is because MagLOV’s singlet state undergoes relatively fast back electron transfer to the ground state, such that higher singlet population leads to more fluorescence. In contrast, the AsLOV2 singlet state forms an FMN-cysteine bond, a long-lived dark state with slow relaxation to the ground state, such that higher singlet population leads to less fluorescence. The same MFE sign therefore reports on different mechanisms underlying the MFE. MagLOV also lacks a strong conformational response, which we confirm with a lack of local change according to cw EPR using an MTSL spin label attached at site K413 (Fig. S8).

Using this understanding of singlet-triplet interconversion for strongly coupled electrons, we constructed a quantum-mechanically informed kinetic model of the AsLOV2 MFE (Fig. 3(c)). The model couples the AsLOV2 photocycle to singlet-triplet interconversion defined by Eq. 2 together with $T_{2}$ relaxation. The state structure is inspired by similar models for MagLOV and previous AsLOV2 work [26, 21, 45]. We fit the MFE field dependence using known g-factors for FMN and cysteine, with D and selected kinetic parameters as free parameters (details in SI S.8). The singlet triplet interconversion is described by a pair of rates $k_{S\to T}$ and $k_{T\to S}$ chosen to reproduce the equilibrium singlet population $\langle P_{S}(t)\rangle =\frac{{\Delta \omega }_{e}^{2}}{2({\Delta \omega }_{e}^{2}+{\left(D∕2+J\right)}^{2})}$. The interconversion rate $\sqrt{{\Delta \omega }_{e}^{2}+{\left(D∕2+J\right)}^{2}}$ is on the order of > 10^8^ Hz, faster than the kinetic decay rates from the radical pair. Because this coherent precession is fast relative to the kinetics, the time-averaged populations capture the relevant dynamics without resolving individual oscillations.

We set J=100MHz as an estimate for the exchange coupling between the radicals, and note that the relevant parameter is D+2J, so a different J rescales D. The resulting fit (Fig. 3(d)) captures the observed field dependence with D=340MHz, matching the most probable value of dipolar coupling across the orientation distribution (found at $\theta =\frac{\pi }{2}$), $D_{⊥}=d∕2=305\;\text{MHz}$, . The exact *g*-factor of cysteine depends on the internal protein hydration state around FMN and the cysteine residue, with simulated and measured values ranging from 2.03 to 2.10 [41, 46], so the *g*-factor used in the simulations may differ from the true value. Relative translational motion of the FMN and cysteine would modulate the inter-radical distance, so that the dipolar coupling would not simply be *d* = 610 MHz as previously defined for a fixed 4.4 Å distance. More importantly, subtle changes in the FMN-cysteine distance by as little as sub-Angstrom can alter the electronic structure in and around FMN, and hence the partial charge distribution and the probability and rate for electron and hydrogen transfer.

The functional shape of the simulation curve was independent of the kinetic parameters, while the field onset of the plateau depended on the spin parameters, and the magnitude depended on both (S9). The ability to capture both shape and magnitude of the fluorescent MFE field dependence with physically reasonable values of *g*, *D*, *J*, and kinetic rates (SI S.8) support our proposed mechanism and shows how strong electron-electron coupling and a Δ*g* effect together produce high-field protein MFEs with strong field dependence.

## 4 Discussion and Conclusion

The FMN-cysteine radical pair in AsLOV2 differs from previously studied photoexcited SCRPs in protein systems. The combination of the radicals’ close spacing and strong coupling with the distinct *g*-factors of cysteine and FMN produces a high-field (> 100 mT) MFE with strong field dependence over the range of 20–1300 mT, reaching 7% at maximum. The same close proximity that creates the strong dipolar coupling also enables FMN-cysteine bond formation from the radical pair, providing a direct route from a quantum spin state to a biochemical signaling state. In this sense, the radical pair functions as a molecular qubit: its singlet and triplet states are distinct, coherently interconvertible, and direct the protein toward distinct biochemical outcomes (bond formation vs. back electron transfer). Conceptually, one can view AsLOV2 as a quantum Arduino, a small embedded device in which a qubit gates a biochemical decision, at ambient temperature, with feedback through its own photocycle, and without an external power supply.

The existence of the MFE provides strong evidence that a spin-correlated radical pair lies on the AsLOV2 photoadduct pathway. The field-dependent singlet-triplet interconversion within a radical pair is the most plausible mechanism of MFE in this system, and the only mechanism proposed to describe the MFEs in flavin systems. This radical pair has been inferred but never directly observed[21, 22], and the strong dipolar and exchange coupling, together with the g-driven mechanism, make it nearly inaccessible to transient EPR, which requires resolvable, weakly coupled spin populations. Direct spectroscopic observation is therefore largely precluded by the very coupling regime that defines the system. The MFE provides the diagnostic that transient EPR cannot: a field effect on both fluorescence and product formation establishes the SCRP without requiring its direct detection. Our unsuccessful AsLOV2 ODMR attempt (S.9) is consistent with this picture rather than at odds with it.

The high-field onset of the AsLOV2 MFE has a methodological implication: protein SCRPs operating in regimes where Δ*g*-driven interconversion dominates over hyperfine-driven interconversion will be invisible at the low-field (< 30 mT) values that dominate the protein MFE literature. Given that LOV proteins are ubiquitous across plants, fungi, bacteria and the human gut microbiome [15], naturally occurring magnetosensitive proteins with high-field MFE may have eluded detection. The fields required to observe AsLOV2-type MFEs are accessible with inexpensive switchable permanent magnets (Fig. S4).

AsLOV2’s SCRP plays a functional role at Earth’s field, where $S_{0}-T\pm$ interconversion is suppressed and bond formation in a triplet-initialized spin state cannot occur prior to decoherence. This creates a natural “quantum barrier” to bond formation, an effect analogous to Pauli spin blockades in double quantum dots [47]. The rate of bond formation is therefore limited by SCRP decoherence, which is sensitive to protein environment and rotational correlation time. AsLOV2 activation kinetics may thus report on hydration and conformational dynamics in a quantum-mechanically constrained regime. Instrumental lifting of this quantum barrier must also be considered when using high fields (i.e. NMR[48, 49]) to monitor LOV kinetics (or other radical termination reactions).

The specific geometry of AsLOV2 is not necessarily optimal for a large MFE at any given field. The field onset and field dependence depend on inter-radical distance and the *g*-factors of the participating radicals, both of which are tunable through site-directed mutagenesis or directed evolution. Designed mutants with weaker dipolar coupling could shift the field onset below 50 mT, where readily generated laboratory fields could enable magnetically tuned bond formation in optogenetic systems [18, 19, 20] or magnetogenetic therapeutic platforms [50, 51]. As with the directed evolution of MagLOV, mutations affect not only the radical pair partners but also the kinetics of the photocycle, such as the electron transfer rate [23, 25]. This engineering space is the design space for biological quantum devices: protein systems in which a single qubit, embedded in a controlled molecular environment, directs a single biochemical decision.

In summary, AsLOV2’s magnetosensitive SCRP links an FMN-cysteine bondforming mechanism directly to a well-characterized protein signaling state. The emergence and field dependence of a previously unseen high-field MFE in AsLOV2 are captured by the interplay of strong dipolar coupling and an unusually low-onset Δ*g* effect. The same magnetic field modulates both the protein fluorescence and the UV–Vis absorption spectrum that reports on FMN-cysteine bond formation preceding conformational activation. This mechanism links coherent spin dynamics to the formation of the FMN-cysteine photoadduct, the bond that gates AsLOV2’s conformational signaling state [29]. This suggests potential applications in optogenetic systems integrating AsLOV2 and in other natural or engineered systems containing a covalent-binding cysteine with a flavin chromophore or similar. The result indicates that protein systems operating in the Δ*g*-driven regime may be more widespread than the prevailing focus on hyperfine-driven MFEs has suggested.

## Supplementary Material

Supplement 1
