# Supplementary material for: Quantum transduction from electron spin state to a signaling state in a wild-type LOV photoreceptor
Source: bioRxiv. 2026 Jul 3:2026.06.30.735402. Preprint. [Version 1] doi: 10.64898/2026.06.30.735402 (PMC13345214; doi:10.64898/2026.06.30.735402)
Supplement: Supplement 1 [file media-1.pdf]

Methods and Supplemental Information: Quantum transduction from electron spin state to a signaling state in a wild-type LOV photoreceptor

William Salvia*[1], Joshua Straub*[1], Shiny Maity[1], Alexey Bogdanov[2], Subhajyoti Chaudhuri[1], Eric Han[1], and Songi Han[1]

[1]Department of Chemistry, Northwestern University, Evanston, IL 60208, USA

[2]Department of Chemical and Biological Physics, Weizmann Institute of Science, Rehovot 7610001, Israel

June 2026

*These authors contributed equally to this work.

Methods

Protein expression, purification, and spin-labeling protocols were derived from Maity et al. 2025 [1].

M.1 Protein expression and purification

DNA fragments encoding the LOV2 domain of *Avena sativa* phototropin 1 (residues 403–546), the MagLOV variant (C450P, D540M, Q513K, L496V, G528K) [2], and K413C mutants of each were purchased from GENE UNIVERSAL or GenScript, cloned into pET-26b(+) via NdeI (5′) and XhoI (3′) restriction sites with a C-terminal 6×His tag. Plasmids were transformed into *E. coli* BL21 (DE3) and plated on LB agar containing kanamycin (0.05 mg/mL). A single colony was used to inoculate 10 mL LB + kanamycin, grown at 37 °C with shaking (220 rpm, 18 h). The primary culture was used to inoculate 1 L TB autoinduction medium containing kanamycin and incubated for ∼16 h at 18 °C in the dark with shaking.

Cells were harvested by centrifugation at 5,000 g for 15 min at 4 °C, resuspended in lysis buffer (20 mM Tris-HCl, 500 mM NaCl, pH 8.0, 5 mM β-mercaptoethanol, 1 mg/mL lysozyme), incubated 30 min at 4 °C, lysed by sonication (60 × 10 s pulses with 10 s intervals), and clarified at 11,000 rpm for 30 min at 4 °C. Lysate was supplemented with 6 mg FMN in 1 mL water (15 min, room temperature, 15 rpm, dark), then combined with 10 mL Ni-NTA resin (2 h, 4 °C, 15 rpm, dark). Resin was washed three times with 40 mL wash buffer (20 mM Tris-HCl, 500 mM NaCl, 40 mM imidazole, 5 mM β-mercaptoethanol, pH 8.0). Protein was eluted with 10 mL elution buffer (as wash buffer, with 500 mM imidazole) and further purified by size-exclusion chromatography (HiLoad™ 16/600 Superdex™ 75 pg, GE Healthcare; NGC™ system, Bio-Rad; SEC buffer: 20 mM Tris-HCl, 150 mM NaCl, pH 8.0). Concentrations were determined by absorbance at 447 nm (AsLOV2) or 450 nm (MagLOV) using $\varepsilon = 13{,}800\ \mathrm{M}^{-1}\ \mathrm{cm}^{-1}$.

M.2 Site-directed spin-labeling

Purified AsLOV2 K413C was reduced overnight at 4 °C with 10 mM DTT, then desalted (PD-10, GE Healthcare). MagLOV K413C does not tolerate overnight DTT exposure and was used without reduction. Both samples were reacted with a 10-fold molar excess of MTSL (1-Oxyl-2,2,5,5-tetramethyl-Δ^3-pyrroline-3-methyl methanesulfonothioate) overnight at 4 °C with gentle shaking in the dark. Excess label was removed by size-exclusion chromatography (HiLoad™ 16/600 Superdex™ 75 pg, equilibrated with 20 mM Tris-HCl, 150 mM NaCl, pH 8.0). Final spin-labeled samples were concentrated to ∼400 μM.

M.3 Inter-radical distances

Inter-radical distances were extracted using ChimeraX-1.10.1 from the AsLOV2 crystal structure (PDB 2v1a) [3]. The AsLOV2 radical pair distance was taken as the FMN C4a to C450 SG distance. The MagLOV radical pair distance was approximated by the FMN C4a to Trp491 CE2 distance on the same structure, since MagLOV's substitutions do not affect the relevant backbone geometry. The AsLOV2 distance is consistent with simulated values from Antill and coworkers [4].

M.4 X-band CW EPR

Continuous wave (CW) EPR spectra were acquired at 293 K on a Bruker EMX X-band spectrometer with a dielectric cavity featuring an optical window (ER 4123D). Samples (10 μL) were loaded into 0.66 mm i.d. quartz capillaries (VitroCom). Photoexcitation used a nominally 450 nm blue laser (peak $\sim$ 460 nm as measured using OceanOptics USB 2000+; nominal power 70 mW, lower at the sample due to optical-window diffraction grating; Laser Components USA). Spectra were recorded in the dark and under continuous 460 nm illumination at a center field of 0.35 T, microwave frequency 9.8 GHz, microwave power 2 mW, modulation amplitude 0.5 G, modulation frequency 100 kHz, sweep width 150 G, averaged over 5 scans.

M.5 X-band fluorescence MFE

Fluorescence measurements were performed at 293 K using a Bruker ElexSys E580 X-band EPR spectrometer and Bruker ER073 electromagnet. Samples (60 μL) in 3 mm quartz EPR tubes (Wilmad) were photoexcited by a 460 nm blue laser (70 mW, Laser Components USA), focused through a Thorlabs F220SMA-532 collimator into a BFH37-600 multimode fiber (600 μm diameter) positioned $\sim$1 cm from the sample. A breadboard between the Helmholtz coils held a 2-inch, 65 mm focal-length plano-convex lens, a Thorlabs 495 nm longpass filter, and a 50 μm Thorlabs fiber connected to a USB 2000+ OceanOptics spectrophotometer. Each data point reflects the fluorescence spectrum acquired in 600 ms; the $480 - 600$ nm region was integrated to quantify fluorescence. The field was cycled on/off in 1-minute periods for 2-min cycles, with 5 cycles per experiment (11 min total), beginning and ending field-off (e.g. 1).

The AsLOV2 MFE vs. field curve (Fig. 3d) was acquired by switching the field intensity during each off cycle in a randomly ordered sequence of 21 field strengths (42 min per run, triplicate). Sample stability over this duration was verified by repeating the 1300 mT measurement for 21 cycles with consistent response.

M.6 Spin dynamics simulation and MFE fitting

The field dependence of the AsLOV2 fluorescence MFE was modeled using a kinetic-model fit with the spin dynamics treated within the S_0–T_0 manifold and

time-averaged.

We used the kinetic scheme shown in Fig. 3(c), which couples the AsLOV2 photocycle to singlet-triplet interconversion within the FMN-cysteine spin-correlated radical pair. The radical pair was initialized in the T_0 state. Triplet-singlet interconversion was modeled as a pair of rates $k_{S \to T}$ and $k_{T \to S}$ chosen to reproduce the time-averaged singlet population

$$\langle P_S \rangle = \frac{\Delta\omega_e^2}{2(\Delta\omega_e^2 + (D_{perp}/2 + J)^2)}, \tag{1}$$

where $\Delta\omega_e$ is the field-dependent Larmor frequency difference between the two radicals, D_{perp} is the most probable value of the dipolar coupling, and J is the exchange coupling. An additional decoherence state $RP1_{dec}$ captured the loss of spin correlation through T_2 relaxation. Simulations were run in MATLAB using *fmincon* constrained nonlinear least-squares minimization routine, with k_{ET}, k_{rec}, R_{1e}, R_2, J, and the g-factors of FMN and cysteine fixed, and k_{ex}, k_{dx}, k_{ISC}, k_{BS}, k_{RP2}, k_f, and D as free parameters. The full rate table and additional fitting details are given in SI S.8.

M.7 Tabletop fluorescence MFE

Tabletop fluorescence measurements were acquired at room temperature using a Magnetpro Multipurpose Switch Magnet (660 lbs lifting strength) clamped to an optical table (Fig. S4). Magnet stability during switching was verified by recording mirror-reflected light intensity over 5 on/off cycles. Field at the magnet surface was ∼300 mT (on) / 40 mT (off), measured with a LATNEX MF-30K gaussmeter. The reflective magnet surface was painted black. Samples (50 μL) in rectangular borosilicate capillaries (VitroCom, 0.4 $\times 4 \times 50$ mm) were photoexcited at 45° with the same 460 nm laser used in M.4, with intensity adjusted to provide fluorescence without heating. Fluorescence collection optics (plano-convex lens, 495 nm longpass filter, 50 μm Thorlabs fiber, USB 2000+ OceanOptics) were identical to M.5; field cycling and quantification were also as in M.5.

M.8 UV–Vis absorption MFE

UV–Vis absorption measurements used a deuterium lamp from a Hamamatsu High Power UV–Vis Fiber Light Source source coupled to a Thorlabs FOFMS-UV In-line fiber filter mount (250-450 nm). The FOFMS-UV was positioned between the poles of the Bruker EMX X-band electromagnet using a 3D-printed holder. A 100 μM AsLOV2 sample in a 1 cm plastic cuvette was illuminated from above by a compact blue laser (70 mW, 460 nm, Laser Components USA) through a BFH37-600 600 μm multimode fiber held in place by a 3D-printed cap. Transmitted light was collected by a 50 μm Thorlabs fiber connected to a USB 2000+ OceanOptics spectrophotometer. Background was acquired with a water-filled cuvette.

Equilibrium experiments. Field cycling matched the fluorescence MFE protocol (1-min on/off, 2-min cycles, 5 cycles, 11 min total). Each data point captured 90 ms of the full 290–550 nm spectrum at 0.4 nm resolution.

Activation and recovery kinetics. Activation was measured by recording 480 nm absorbance following onset of blue-light excitation; recovery was measured following light shutoff after 60 s of irradiation. Each data point captured 90 ms of absorbance. Field-on and field-off experiments used one undisturbed sample, with experiment order randomized. Activation time courses were fit to a single exponential decay,

$$A(t) = b + A_0 e^{-k_{act}t}, \tag{2}$$

where $A(t)$ is the absorbance at time t, A_0 is the absorbance change between dark and lit states, b is the baseline absorbance at $t \to \infty$, and k_{act} is the activation rate. Recovery time courses were fit to

$$A(t) = A_{eq}(1 - e^{-k_{rec}t}), \tag{3}$$

where A_{eq} is the equilibrium dark-state absorbance and k_{rec} is the recovery rate. Each condition was measured across 6 independent trials. Statistical significance was assessed by paired t-test (5 degrees of freedom). The 650 mT recovery rate difference Δk_{rec} was $-0.15 \pm 0.32\%$.

M.9 MFE quantification

Fluorescence MFE was defined as $\Delta I / I_{off}$, where I is the fluorescence intensity and I_{off} is the field-off intensity from a background spline fit to the field-off periods (Fig. S1). For noisier datasets, a 3–5 point (1.8–3 s) moving average was applied before spline fitting. UV–Vis MFE (Fig. 2(b-d) at wavelength λ was defined analogously as $\Delta A / A_{off}$. For Fig. 2(e-f), the percent change in UV–Vis activation and recovery time constants ($\Delta\tau$) was calculated as

$$\Delta\tau = \frac{\tau_{off} - \tau_{on}}{\tau_{off}}. \tag{4}$$

Error bars on the on and off rates were 95% confidence intervals (t-test, 5 degrees of freedom), and error in $\Delta\tau$ was propagated as

$$\sigma_{\Delta\tau} = \sqrt{\left(\frac{\sigma_{on}}{\tau_{off}}\right)^2 + \frac{(\tau_{on} \cdot \sigma_{off})^2}{\tau_{off}^4}}. \tag{5}$$

Spline-fitting details are in SI S.1.

M.10 Optically detected magnetic resonance

ODMR was acquired on a Bruker EMX X-band spectrometer with a Bridge 12 microwave power source (up to 10 W). MagLOV samples (100 μM in 3 mm capillaries) were placed in a tuned cylindrical dielectric resonator. The fluorescence

collection optics matched M.5 (plano-convex lens, 495 nm longpass, 50 μm fiber, USB 2000+). The field was swept 3254.4–3454.4 G in 66 s with microwaves at 9.407 GHz, 36 dBm (4 W). Fluorescence acquisition was synchronized with microwave onset and sweep start. MagLOV was not temperature-controlled. AsLOV2 ODMR (500 μM) was attempted at 4 °C using a Variable Temperature Unit, sweeping 2920–3720 G in 300 s at 9.80 GHz with powers between 4 and 10 W; no measurable contrast was observed (see SI S.9).

Supplementary Information

S.1 Spline fit for MFEs in fluorescence and UV–Vis data

Using fluorescence spectra from the OceanOptics spectrometer, we integrated the region between 480 and 600 nm at each timepoint. We then plotted these integral values vs. time. To control for baseline drift and initial saturation period in AsLOV2 and MagLOV magnetic field effects, we implemented a background spline fit using manually selected points at the final field off point before the field was turned on for each cycle, following Abrahams et al.[5]. The background spline fit was then subtracted from the experimental data, and the processed data was plotted as described in section M.9. The steps in this processing are shown below for an AsLOV2 fluorescence MFE dataset taken at 650 mT.

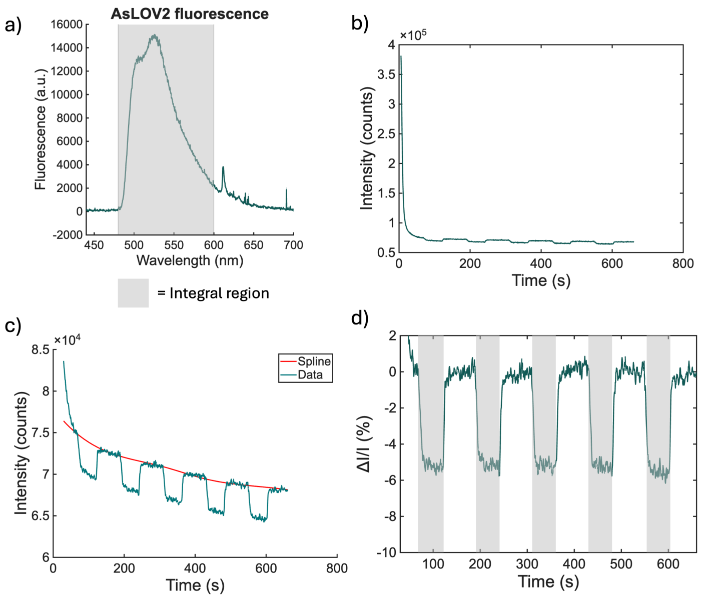

Figure S1: Processing of MFE data. **(a)** AsLOV2 fluorescence spectrum under blue light illumination, with integration region marked in grey. **(b)** Raw integrated fluorescence counts. **(c)** Spline fit to the background (field off) fluorescence. **(d)** Processed MFE data, plotted as $\Delta I/I_{off}$, with field on regions marked in grey.

S.2 X-band optical setup for fluorescence MFE

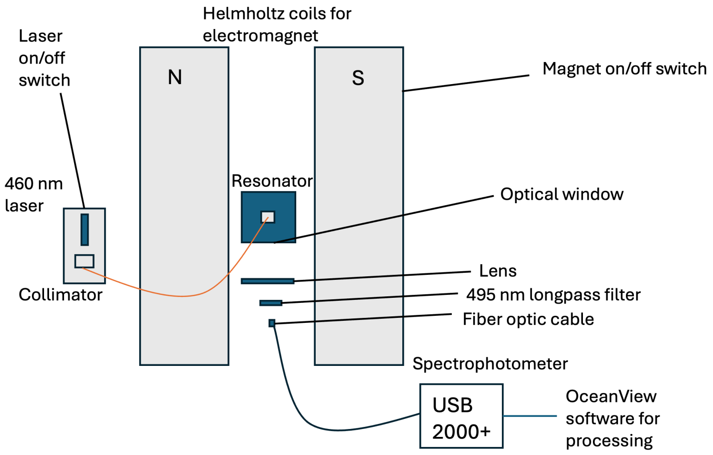

Figure S2: Setup used for fluorescence MFE measurements on X-band spectrometer with electromagnet as viewed from above.

S.3 FMN control experiment

To confirm that the observed fluorescence MFE in MagLOV and AsLOV2 for our setup was from the magnetically altered fluorescence of the protein, we conducted a control experiment on FMN alone. Without an electron donor in the sample, FMN should not show any magnetic field effects on its fluorescence. We thus ran a MFE experiment on a sample of 400 μM FMN at 1300 mT for three field on-off cycles following the same protocol as used for the AsLOV2 and MagLOV samples. The data is shown below in Fig. S3, and shows no MFE for FMN. Note that the fluctuations in the intensity are not periodic with magnetic field, and are likely from sample diffusion with respect to the beam path.

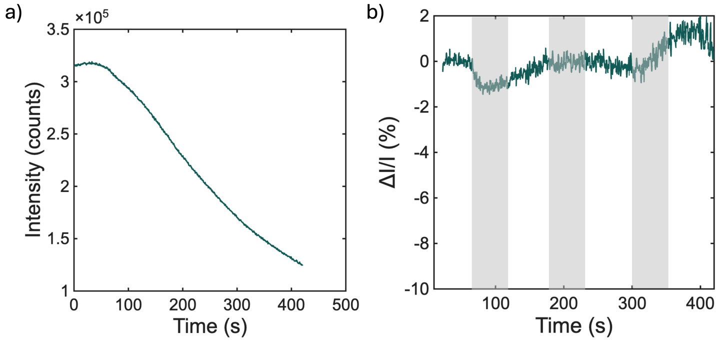

Figure S3: Fluorescent MFE control on 400 μM FMN at 1300 mT. **(a)** Raw data for FMN fluorescence MFE. **(b)** Processed data after fitting a spline based on the timing of the field on/off cycles.

S.4 Tabletop optical setup for fluorescence MFE

The AsLOV2 MFE is accessible for experimental study without the need for high-field electromagnets. On a second tabletop setup, we used a switchable magnet (see M.7 for details), which can be purchased for \$60 on Amazon, to observe the AsLOV2 MFE. The magnitude of the MFE on this setup was $\sim$3% at 300 mT, a comparable size to the same field on the X-band fluorescence set-up using an electromagnet.

To control for any systematic error from magnet field switching, we secured a 1.5 inch thick wooden block to the front of the magnet to reduce the field strength experienced by the same AsLOV2 sample to 40 mT as measured by the gaussmeter. If the change in fluorescence was due to factors such as changed sample position, we would expect a change in the fluorescence even at this low field value. No change was observed.

An additional control was an 400 μM FMN sample in buffer (20 mM TRIS, 150 mM NaCl, pH 8.0), expected to have no MFE. When placed flush against the magnet, no MFE was observed at 300 mT.

These experiments establish that AsLOV2's MFE is reproducible on different setups. This is a low-budget setup ($<$ \$1000 total) for others interested in studying AsLOV2-like MFEs using fluorescence.

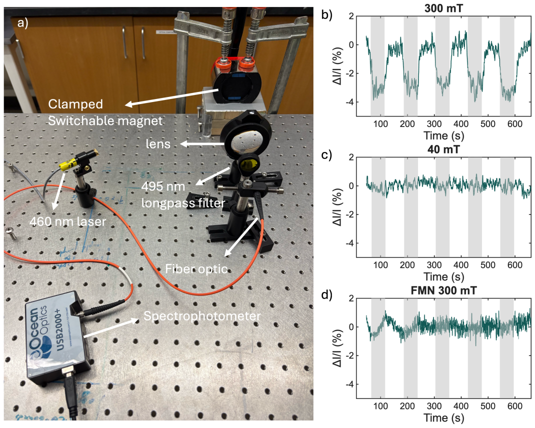

Figure S4: Tabletop MFE setup. **(a)** Picture of setup used to record table fluorescence MFE. **(b)** AsLOV2 fluorescence MFE when the sample is directly on the magnet, generating a field of $\sim$300 mT for the magnet on period and $\sim$40 mT for the magnet off period. The magnitude of the MFE agrees with the value measured at 300 mT on the setup using the electromagnet. **(c)** AsLOV2 fluorescence MFE when the a wooden block is placed between the magnet and the sample, so that the field on period has a magnetic field of $\sim$40 mT and the field off period has roughly zero field. **(d)** FMN fluorescence MFE with a 400 μM FMN sample placed directly on the magnet. For (b)-(d), grey regions indicated periods of field on.

S.5 UV–Vis absorption MFE setup

For the absorption MFE experiment at an equilibrium between cysteine bound and unbound AsLOV2, we illuminated the sample using the setup showed in Fig. S5 such that the photoactivation rate matched the recovery rate and the UV-Visible spectrum remained at a steady-state, with visible cysteine-unbound absorption peaks at 447 and 473 nm (Fig. S6).

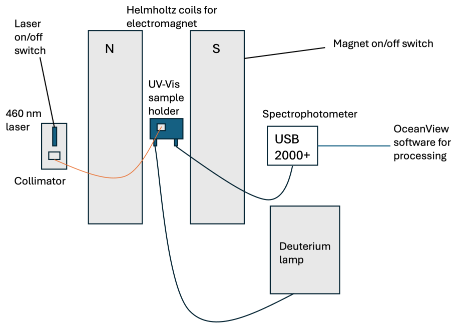

Figure S5: Setup for UV-Visible absorption MFE measurements as viewed from above.

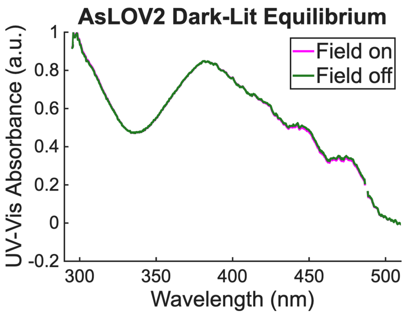

Figure S6: UV–Vis absorption spectrum under equilibrium conditions for both field on (650 mT) and field off conditions. A comparison to the spectrum shown in Fig. 2(a) shows that features of both the dark and lit state are present. The difference between these spectra is 2(b)

.

S.6 UV–Vis absorption MFE at 350 mT

We used the same protocol as the 650 mT experiment, except that we positioned the UV–Vis sample holder at a spot in the X-band magnet such that the field at the sample was weaker (350 mT). UV–Vis absorption was then measured at 480 nm as a function of time after the laser was initially turned on (activation), or after the laser was turned off following 60 s of irradiation (recovery) over 9 experiments with the same untouched sample. The first experiment had a discontinuity due to a shift in the cuvette position, so it was neglected. The average time constant of activation, fit to a monoexponential (see main text for details), is significantly different with field off/on: 2.60 ± 0.05 s with the field on and 2.52 ± 0.03 s with the field off ($p = 0.002$) (Fig. S7(a,b)). This is a 3.1 $\pm$ 2.0 % MFE for the time constants, using the mean and error equations from Methods M.8. This value is consistent with the fluorescent MFE at 350 mT (Fig. 3(d)). The differences in activation time constant between 350 mT and 650 mT datasets are due to different setup conditions, since the activation rate of AsLOV2 is highly dependent on the intensity of laser light for photoactivation, and the laser and collimator was positioned differently between days.

Some drift in the first two experiments, likely due to the sample shifting, led to these first two experiments being outliers in the recovery data. Including those two experiments, the difference between field on and field off recovery time constants was -0.7 ± 2.2 %, non-significant ($p = 0.11$) (Fig. S7(c,d). Removing these two outliers, the difference between field on and field off recovery time constants was -0.18 ± 0.61 %, still non-significant ($p = 0.32$) (Fig. S7 (e,f)). We processed it both ways to visually demonstrate below that the recovery rate is not affected. Removing the same two data points does not have a significant effect on the activation time constants under field off and on conditions (mean on: 2.52 s, mean off: 2.62 s, a difference of 3.8 $\pm$ 2.9 %). A significant activation magnetic field effect and non-significant recovery effect is consistent with the 650 mT data (Fig. 2) and the fluorescence MFE size (Fig. 2(d)).

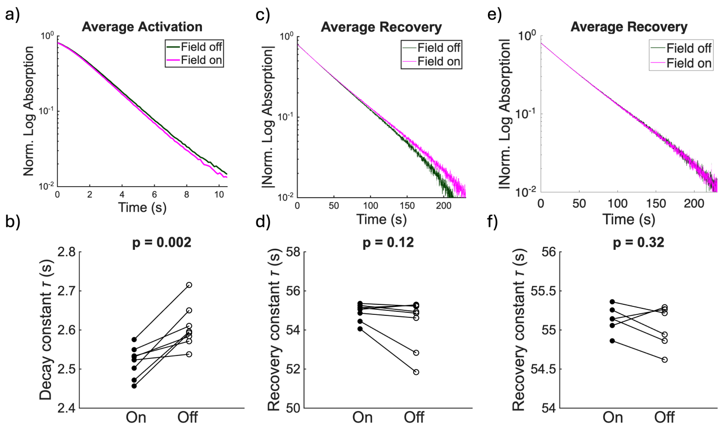

Figure S7: UV–Vis absorption MFE at 350 mT. **(a)** Average activation for field on and field off. **(b)** Decay constant for each trial and corresponding p-value for a paired t-test between field on and field off trials. **(c)** Average recovery for field on and field off. **(d)** Recovery constant for each trial and corresponding p-value for a paired t-test between field on and field off trials. **(e)** Average recovery for field on and field off excluding the first two trials. **(f)** Recovery constant for each trial and corresponding p-value for a paired t-test between field on and field off trials excluding the first two trials.

S.7 Conformational response of AsLOV2 and MagLOV

By adding an MTSL spin label to AsLOV2 with site K413 mutated to a cysteine, we noticed that it exhibits a very large change in X-band CW EPR lineshape compared to previous work (compare to less-responsive spin-labeled positions in Maity et al. 2023[6] and Maity et al. 2025[1]). This large change in lineshape is indicative of increased conformational restriction of the spin label upon protein conformational change by a Microscopic Order Macroscopic Disorder (MOMD) model[7]. We consider this change in lineshape to be a proxy for the unique AsLOV2 unfolding mechanism that can be monitored at room temperature with a standard X-band EPR spectrometer. When MagLOV is labeled at the same site, it has a very similar spectrum in the dark state. However, we do not see a change in lineshape upon photoactivation like that of wild-type AsLOV2's. The observed change is likely due to a baseline feature that likely corresponds to MagLOV's photobleaching and subsequent aggregation. These data indicate that MagLOV does not respond to light with a conformational response like AsLOV2's. This is not a trivial result despite MagLOV's lack of the flavin-binding cysteine, since LOV domain VIVID is known to enter a signaling state even when the cysteine is removed[8].

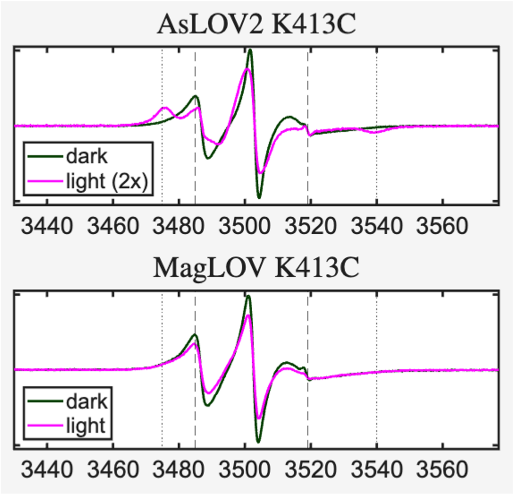

Figure S8: Comparison of K413C mutated AsLOV2 and MagLOV CW EPR spectrum for light and dark states. The light state spectrum for AsLOV2 K413C is scaled 2-fold so that the lineshape is easily compared to the dark state.

S.8 Simulation of AsLOV2 fluorescence MFE

The kinetic model used to fit the AsLOV2 fluorescence MFE is shown in Fig. 3(c). Blue-light excitation of the FMN ground state generates a singlet excited state, which undergoes intersystem crossing to a triplet excited state. Electron transfer from C450 to FMN forms the spin-correlated radical pair (RP1) in the T_0 state, with magnetic field–dependent singlet-triplet interconversion governed by Eq. 2. The decoherence state $RP1_{dec}$ captures the loss of spin correlation through T_2 relaxation. Singlet RP1 (and $RP1_{dec}$) decay through back electron transfer via RP2 (returning to the FMN ground state) or covalent FMN-cysteine bond formation (yielding the long-lived signaling state, which slowly recovers to the ground state).

We initialize the radical pair in the T_0 state. Following intersystem crossing the triplet is spin-polarized with unknown populations of T_-, T_0, and T_+, but the $T_\pm$ states never efficiently interconvert with S_0: at low fields interconversion is blocked by the strong dipolar coupling, and at high fields by the large Zeeman energy difference. Since T_{1e} is expected to exceed T_2 for the radical pair, we assume negligible mixing of the triplet states during the SCRP lifetime. Different initial triplet polarizations are absorbed into the rate of SCRP formation in the model.

We fit the experimental MFE using this model with k_{ET}, k_{rec}, R_{1e}, R_2, J, and the g-factors for FMN and cysteine fixed, and k_{ex}, k_{dx}, k_{ISC}, k_{BS}, k_{RP2}, k_f, and D as free parameters. Constraints were placed on the ground state population based on the degree of fluorescence saturation observed. The rates

for singlet-triplet interconversion were set to establish the equilibrium singlet population $\langle P_S(t)\rangle = \Delta\omega_e^2/2(\Delta\omega_e^2 + (D/2 + J)^2)$ within 4 ns, given that the timescale for singlet-triplet interconversion is at minimum $D/2 + J > 250$ MHz, as described in the main text. The simulation shown in Fig. 3(d), resulting from the best fit to the experimental data, used the parameters listed in Table 1.

Table 1: Best-fit rate constants and spin parameters for the AsLOV2 fluorescence MFE simulation. Rates and D, J, R_{1e}, R_2 in Hz; g-factors dimensionless.

Parameter	Value
k_{ex}	101
k_{dx}	7.23×10^{10}
k_{ISC}	5.745×10^{8}
k_{ET}	2×10^{8}
k_{RP2}	9.33×10^{4}
k_f	1.0×10^{6}
k_{BS}	2.7×10^{6}
k_{rec}	0.02
$g_{FMN,iso}$	2.0034
$g_{cyst,iso}$	2.035
R_{1e}	1×10^{3}
R_2	1×10^{6}
D	680×10^{6}
J	100×10^{6}

Note that the shape of the simulation as a function of field is determined primarily by the spin parameters, while the kinetic rates determine the exact magnitude of the effect. To demonstrate this, we fixed the spin parameters shown in Table 1, and randomly varied the kinetic parameters by a scaling factor between 0.1 and 10 (an order of magnitude lower or higher) and calculated the resulting MFE. The results are shown in Fig. S9, with the shape of the MFE curve conserved across the different sets of kinetic parameters, while the magnitude of the effect is highly sensitive to the kinetic parameters.

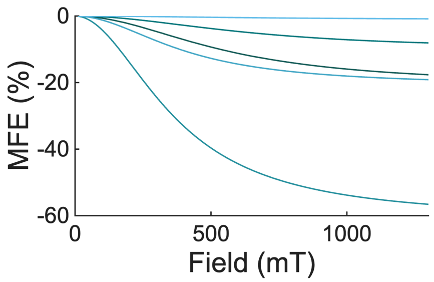

Figure S9: MFE simulations varying kinetic parameters while keeping spin parameters fixed. For each of the five curves, the kinetic rates were independently and randomly scaled up to an order of magnitude higher or lower.

S.9 Optically Detected Magnetic Resonance (ODMR)

Following Abrahams et al. [5], we replicated their MagLOV cw-ODMR spectrum at X-band fields (see Methods for details). Note that our spectrum is significantly broader because of the high microwave power used on our setup. We attempted to do ODMR on AsLOV2, but attempts were unsuccessful (no contrast was observed), likely due to a broad spectrum due to broadening from the dipolar and exchange couplings and highly temperature-dependent fluorescence obscuring any genuine signal. Future work with AsLOV2 cw-ODMR will likely require cryogenic conditions and longer experiment times.

Additionally, cw-ODMR of AsLOV2 would function differently from MagLOV due to the different mechanisms leading to the MFE for each protein. In MagLOV, the presence of a field generally decouples the $T_{\pm}$ levels from the S_0 state, and introducing microwaves recouples the $T_{\pm}$ levels to the T_0 level and thus the S_0 state, leading to a recovery of the fluorescence decrease seen in the field on conditions without microwaves. However, in AsLOV2 the $T_{\pm}$ levels are never coupled to the S_0 state in either the field on or field off conditions. When the field is off, the triplet and singlet states are eigenstates of the spin Hamiltonian, as described in the main text, and no interconversion occurs. When the field is sufficiently high to allow for $T_0 - S_0$ interconversion, the Zeeman energy of the $T_{\pm}$ states decouples these states from interconversion with S_0. While microwaves could couple the $T_{\pm}$ and T_0 states, the effect on the AsLOV2 fluorescence is not clear as this does not recover the initial field off behavior as in the case of MagLOV.

See the Methods section for more details about the attempted experiments.

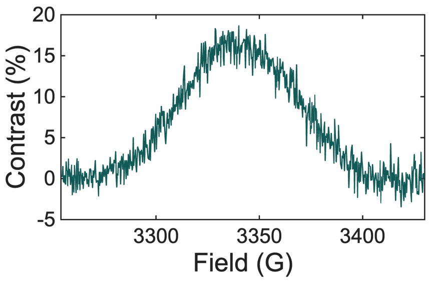

Figure S10: MagLOV ODMR spectrum at X-band. A background function was fit and subtracted from the spectrum to account for drift in fluorescence from sample heating during the experiment.

S.10 TDDFT Quantum Chemistry Calculations

The magnetic field effect (MFE) reported in the main text requires a photogenerated, spin-correlated FMN-cysteine radical pair (SCRP) on the photoadduct pathway, formed by electron transfer from C450 to the photoexcited flavin. The MFE itself is diagnostic of this radical pair, but the strong dipolar and exchange coupling that set the high-field onset also preclude its direct observation by transient EPR (main text and S.9). To provide an independent, structure-based check on the two photochemical premises that underlie the kinetic model of Fig. 3(c) - (i) that the dark and lit states carry the distinct UV–Vis signatures used as the MFE readout in Fig. 2, and (ii) that low-lying flavin→cysteine charge-transfer (CT) states are available to seed the electron-transfer step, we carried out time-dependent density functional theory (TDDFT) on a truncated FMN-cysteine dyad in implicit water.

Single-point TDDFT calculations were performed in Q-Chem 6.3 at the ωB97X-D/def2-TZVP level, with the linear-response formalism including both singlet and triplet roots (10 roots per calculation). Bulk aqueous solvation was modeled with the polarizable continuum model (PCM, $\varepsilon = 80$). Charge-transfer character was quantified with the built-in CT metrics (NTO-based spatial overlap Λ and charge-displacement length Δr), and natural transition orbitals were computed for the leading transitions.

Two chromophore models were used. The **dark** model comprises the oxidized lumiflavin-truncated isoalloxazine ring with a non-covalently associated cysteine fragment bearing the reactive thiol. The **lit** model uses the reduced/ protonated flavin (additional proton in the N5 region, as in the neutral flavin photoproduct) with the same cysteine fragment covalently bound to flavin. For each model the closed-shell singlet ground-state surface (charge 0, multiplicity 1) gives the optical absorption, while a separate unrestricted triplet reference (charge 0, multiplicity 3; $\langle S^2 \rangle \approx 2.02$–$2.03$, indicating negligible spin contamination) characterizes the photoexcited triplet manifold that precedes radical-pair formation. The flavin was modeled without the ribityl phosphate tail and an optimized geometry was used; absolute transition energies are therefore approximate and the analysis relies on relative trends and state characters, which are robust to these truncations.

Figure S11 shows the broadened singlet absorption spectra for the dark and lit models, normalized to their respective maxima and plotted on the same axes and color convention as Fig. 2(a) (dark, green; lit, magenta). Each spectrum was generated by convolving the optically allowed singlet roots with Gaussians of 0.30 eV FWHM in the energy domain. Triplet roots are optically dark from the singlet ground state and are excluded from the absorption envelope.

The dominant bright transition of the dark model is a flavin-localized $\pi\pi^*$ excitation at 3.43 eV (362 nm, $f = 0.44$), dominated by a single HOMO→LUMO transition with small charge displacement ($\Delta r_{\text{NTO}} = 0.76$ Å, $\Lambda = 0.70$). In the lit model the corresponding bright band shifts to the blue, to 3.84 eV (323 nm, $f = 0.30$). This blue shift and loss of oscillator strength upon conversion to the reduced/bound flavin reproduces the direction of the experimental dark→lit

change in Fig. 2(a), in which the resolved dark-state bands at 447 and 473 nm collapse and intensity migrates to ∼380 nm and below 300 nm. The computed band positions are uniformly blue-shifted relative to experiment by ∼0.4–0.5 eV, as expected for a truncated isoalloxazine with a range-separated hybrid functional; the calculations are therefore corroborative of the *assignment* in Fig. 2(a) rather than a quantitative reproduction of the band maxima.

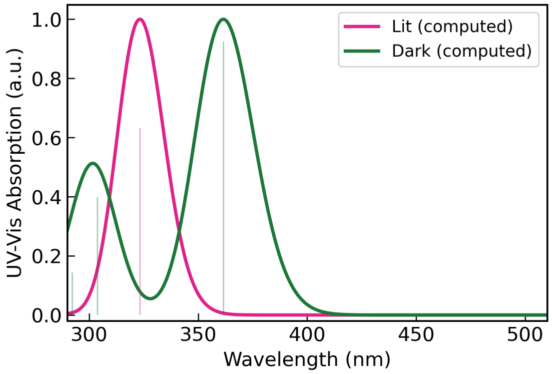

Figure S11: Computed TDDFT singlet absorption spectra of the FMN-cysteine dyad in implicit water (ωB97X-D/def2-TZVP, PCM ε = 80). Dark model (oxidized flavin) in green; lit model (reduced/protonated flavin) in magenta. Vertical sticks mark the underlying singlet oscillator strengths. The bright flavin $\pi\pi^*$ band shifts to the blue and loses intensity on going from the dark to the lit state, reproducing the direction of the experimental dark→lit change in Fig. 2(a). Absolute band positions are blue-shifted relative to experiment due to the truncated chromophore model and the functional.

References

[1] Shiny Maity, Jackson Sheppard, Hannah Russell, Chung-Ta Han, Leah Epstein, Bruce A. Johnson, Brad D. Price, Ruixian Han, Lokeswara Rao Potnuru, Jinlei Cui, Mark S. Sherwin, Joan-Emma Shea, Janet E. Lovett, Kevin H. Gardner, and Songi Han. Hydraulic Activation of the AsLOV2 photoreceptor, June 2025.

[2] Rebecca Frank Hayward, Ashleen Rai, Julia R. Lazzari-Dean, Austin E.Y.T. Lefebvre, Andrew G. York, and Maria Ingaramo. Magnetic control of the brightness of fluorescent proteins, May 2024.

[3] Andrei S. Halavaty and Keith Moffat. N- and C-Terminal Flanking Regions Modulate Light-Induced Signal Transduction in the LOV2 Domain of the Blue Light Sensor Phototropin 1 from Avena sativa,. *Biochemistry*, 46(49):14001–14009, December 2007.

[4] Lewis M. Antill, Joseph Baidoo, and Luca Gerhards. Revealing properties for enhanced quantum sensing in engineered proteins, March 2026. ISSN: 2692-8205.

[5] Gabriel Abrahams, Ana Štuhec, Vincent Spreng, Robin Henry, Idris Kempf, Jessica James, Kirill Sechkar, Scott Stacey, Vicente Trelles-Fernandez, Lewis M. Antill, Christiane R. Timmel, Jack J. Miller, Maria Ingaramo, Andrew G. York, Jean-Philippe Tetienne, and Harrison Steel. Quantum spin resonance in engineered proteins for multimodal sensing. *Nature*, 649(8099):1172–1179, January 2026.

[6] Shiny Maity, Brad D. Price, C. Blake Wilson, Arnab Mukherjee, Matthieu Starck, David Parker, Maxwell Z. Wilson, Janet E. Lovett, Songi Han, and Mark S. Sherwin. Triggered Functional Dynamics of AsLOV2 by Time-Resolved Electron Paramagnetic Resonance at High Magnetic Fields. *Angewandte Chemie International Edition*, 62(13):e202212832, 2023. _eprint: https://onlinelibrary.wiley.com/doi/pdf/10.1002/anie.202212832.

[7] Christian Altenbach and David Budil. Analyzing CW EPR Spectra of Nitroxide Labeled Macromolecules. *Applied Magnetic Resonance*, 55(1):159–186, March 2024.

[8] Estella F. Yee, Ralph P. Diensthuber, Anand T. Vaidya, Peter P. Borbat, Christopher Engelhard, Jack H. Freed, Robert Bittl, Andreas Möglich, and Brian R. Crane. Signal transduction in light–oxygen–voltage receptors lacking the adduct-forming cysteine residue. *Nature Communications*, 6(1):10079, December 2015.
